# The complete chloroplast genome of the medicinally important plant *Plumbago zeylanica* L. (plumbaginaceae) and phylogenetic analysis

**DOI:** 10.1080/23802359.2024.2333574

**Published:** 2024-04-03

**Authors:** Hao Zhou, Huajie Zhang

**Affiliations:** a Key Laboratory of Molecular Biophysics of the Ministry of Education, College of Life Science and Technology, Huazhong University of Science and Technology, Wuhan, China; b CAS Key Laboratory of Plant Germplasm Enhancement and Specialty Agriculture, Wuhan Botanical Garden, Chinese Academy of Sciences, Wuhan, Hubei, China; c Center of Conservation Biology, Core Botanical Gardens, Chinese Academy of Sciences, Wuhan, Hubei, China; d University of Chinese Academy of Sciences, Beijing, China

**Keywords:** Complete chloroplast genome, phylogenetic analysis, *Plumbago zeylanica* (plumbaginaceae)

## Abstract

*Plumbago zeylanica* L. 1753 is a medicinally-important herb in family Plumbaginaceae. In this study, we assembled and reported the complete chloroplast genome of *P. zeylanica*. The plastome of *P. zeylanica* was 169,178 bp, including a large single-copy region of 92,135 bp, a small single-copy region (SSC) of 13,455 bp and a pair of inverted repeat regions (IRs) of 31,794 bp. It contained 124 genes, including 79 protein-coding genes, 37 tRNA genes and eight rRNA genes. Phylogenetic analysis showed that *P. zeylanica* formed a close relationship with *P. auriculata* in *Plumbago.* The first complete chloroplast genome report of *P. zeylanica* providing an opportunity to explore the genetic diversity, and would be also helpful in the species identification and conservation.

## 1. Introduction

**Figure 1.**
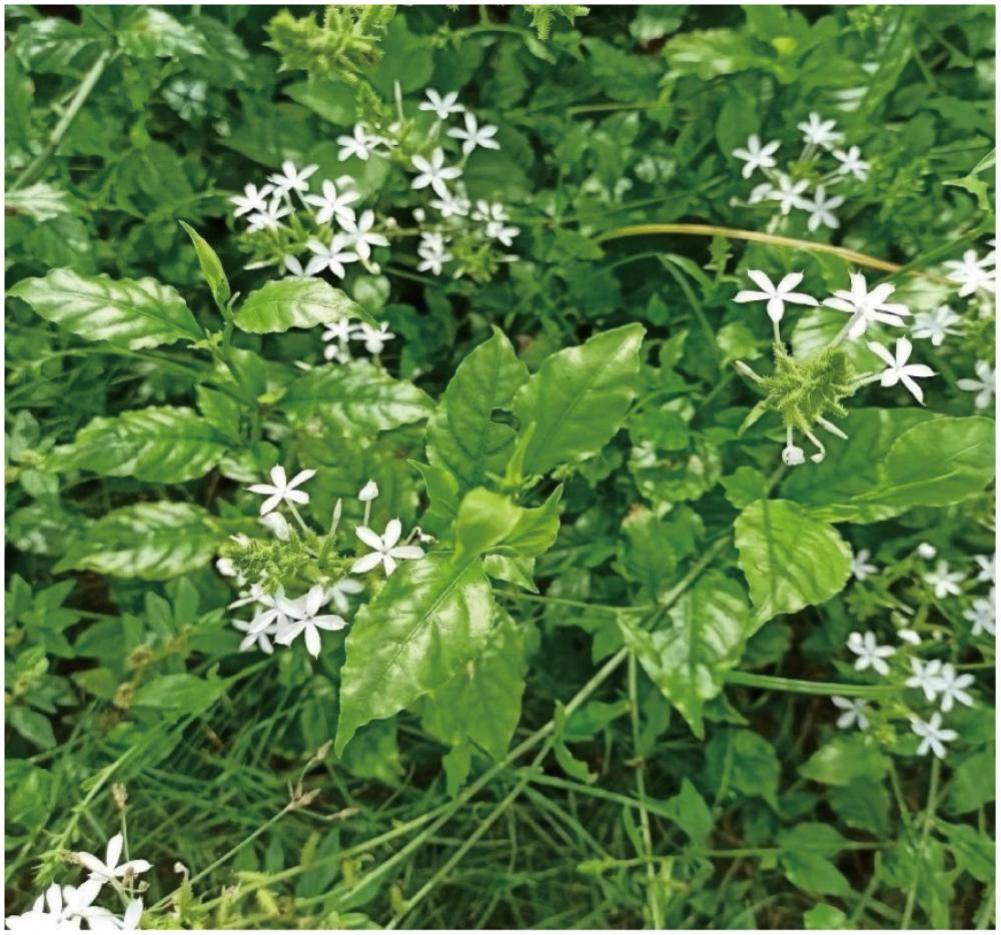
Morphology of plants and habitat map of *P. zeylanica* from baxian village, pingji town, qinzhou city, Guangxi Province, China (22°9′27″ N; 108°44′53″ E). Morphological characteristics: Perennial herbs or shrubs; branches spreading, often lianous; leaf blade ovate, base cuneate to obtuse, apex acuminate and mucronate; inflorescences spicate-racemose, bracts subovate, apex acuminate; corolla white to pale bluish white; capsules pale yellow-brown, oblong. Photograph by Huajie Zhang.

*Plumbago* species are mainly distributed in warm tropical regions of the world. Three species within the genus, including *Plumbago indica* L. 1754, *Plumbago zeylanica* L. 1753 and *Plumbago auriculata* Lam. 1786, are important medicinal plants and are widely cultivated around the world (Sandeep et al. 2011; Koutroumpa et al. 2018). *P. zeylanica*, commonly known as white chitrak, is a perennial herbs or shrubs in Plumbaginaceae (Figure 1). It is supposed to be originated in South-East Asia and is one of the medicinal plants used in the Indian and China traditional system of medicine (Sheeja et al. 2010). Plumbagin is a highly potent and broad-spectrum biological compound, and it is mainly extracted from the root and leaves of *P. zeylanica* (Yuvaraj and Jalalpure 2011; Adusei et al. 2019; Choudhary et al. 2021). Plumbagin displays various potential medicinal properties such as anti-cancer, anti-fungal, anti-inflammatory, anti-bacterial, anti-fertility, anti-malarial and Antidiabetic (Sheeja et al. 2010; Choudhary et al. 2021).

*Plumbago zeylanica* is the most commonly cultivated and utilized species in *Plumbago* for its medicinal and pharmacological properties (Checker et al. 2009; Shukla et al. 2021). However, the complete chloroplast genome sequence of *P. zeylanica* has not been reported. At present, the phylogeny of *Plumbago* is still not clear due to the incomplete sampling and fragment markers providing insufficient informative loci (Koutroumpa et al. 2018). We expect the complete chloroplast genome sequence of *P. zeylanica* will benefit the phylogenetic analysis of *Plumbago* and Plumbaginaceae and believed that it will helps to explore the genetic diversity and population structure of *P. zeylanica.*

## 2. Materials and methods

The sample of *P. zeylanica* was collected from Guangxi Province of China (22°9′27″ N; 108°44′53″ E) in September 2023. The specimen was deposited in Wuhan botanical Garden with the specific identifying number of BHD-2023 (contacts: Guangwan Hu, guangwanhu@wbgcas.cn). Total genomic DNA was extracted from dried leaves, then sequenced using Illumina Hiseq Platform (San Diego, CA, USA). Finally, more than 5 GB of raw sequence data were obtained. The chloroplast genome of *P. zeylanica* was assembled with NOVOPlasty v4.3.1(Dierckxsens et al. 2017) with the plastome sequence of *P. auriculata* (NCBI accession NC041245) as the reference (Yao et al. 2019). The genome annotation was performed with Geneious v9.0.2(Kearse et al. 2012) and Geseq (https://chlorobox.mpimp‐golm.mpg.de/geseq.html). The circular plastid genome map was completed using the online program OGDRAW (https://chlorobox.mpimp-golm.mpg.de/OGDraw.html). The complete chloroplast genome sequence of *P. zeylanica* was submitted to Genbank with the number OR712438. Simple Sequence Repeats (SSRs) were detected in MISA (Beier et al. 2017) with default parameters.

A total of 13 plastome sequences, including 11 from Plumbaginaceae, were considered as ingroups for phylogenetic analysis. The complete plastome sequences of *Plumbago zeylanica* and the other ten representative species from Plumbaginaceae were downloaded from GenBank. We take *Rheum alexandrae* (MZ997426) and *Polygonum aviculare* (OK661156) from the Polygonaceae (which is sister to Plumbaginaceae) as outgroups (Zhang et al. 2022a; 2022b). The complete chloroplast genome sequence that removes one repeat of IR region were aligned with MAFFT v7.5(Katoh and Standley 2013). We conducted the maximum-likelihood (ML) analysis with raxmlHPC-PTHREADS (Stamatakis 2014), and 1000 rapid bootstrap replicates were conducted.

## 3. Results

**Figure 2.**
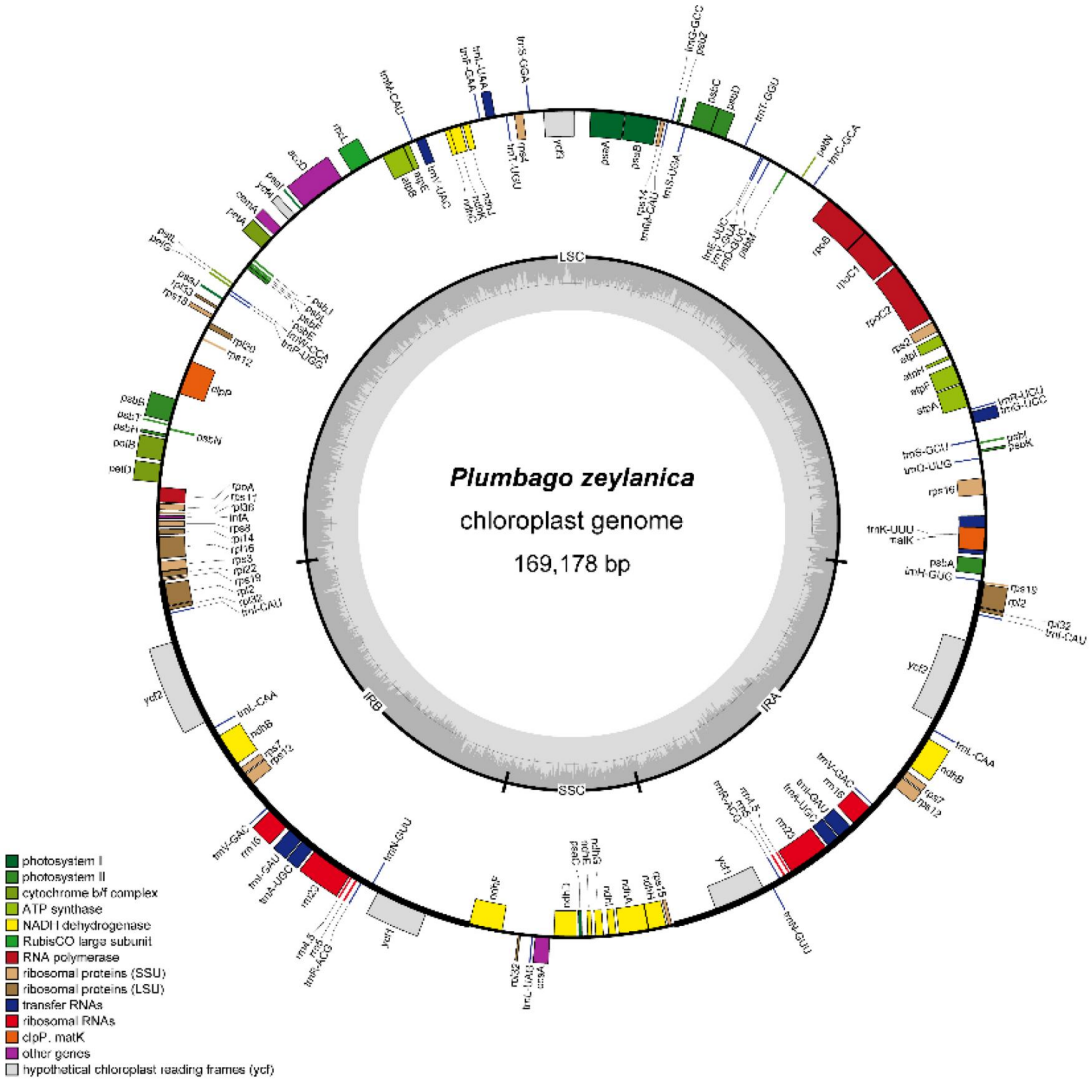
Circular map of the *P. zeylanica* chloroplast genome. Genes shown inside the circle are transcribed clockwise, those outside the circle are counterclockwise transcribed. The light grey and the darker grey in the inner circle represent at and GC content, respectively. Different functional groups of genes are signed according to the colored boxes. LSC: large single copy; SSC: small single copy; IRA/IRB: Inverted repeat regions.

**Figure 3.**
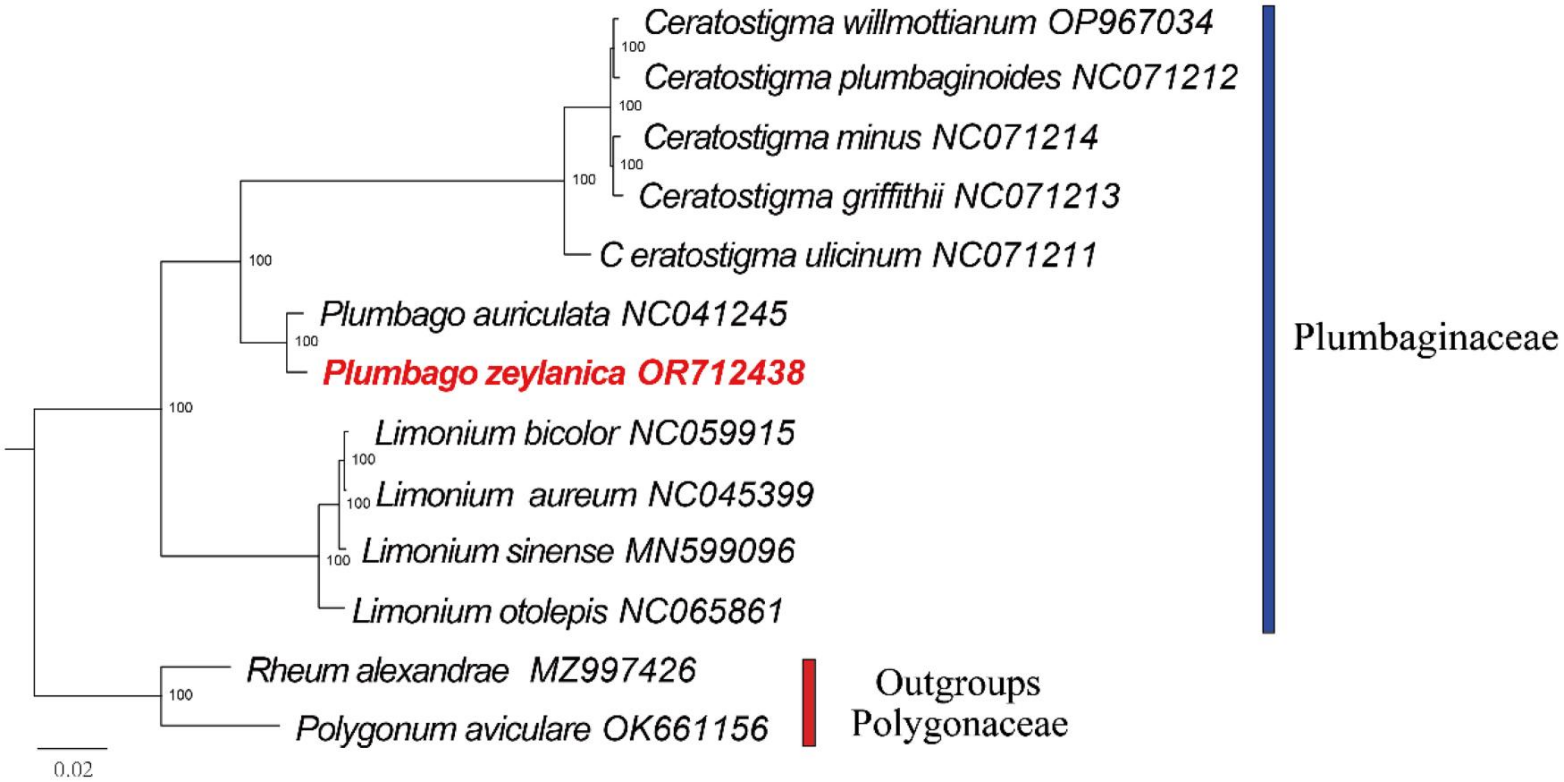
Maximum-likelihood (ML) phylogenetic tree of plumbaginaceae species based on the concatenated LSC, SSR and an IR of chloroplast genome. The numbers in nodes indicate the bootstrap support values. The red fonts represent *R. idaeus* in this study. The clades of species are represented with the bar charts. Two species (*rheum alexandrae* and *polygonum aviculare*) in polygonaceae were selected as outgroups. The following sequences were used: *Ceratostigma willmottianum* OP967034 (Zhao et al. 2023), *ceratostigma plumbaginoides* NC071212 (Zhao et al. 2023), *ceratostigma minus* NC071214 (Zhao et al. 2023), *ceratostigma griffithii* NC071213 (Zhao et al. 2023), *ceratostigma ulicinum* NC071211 (Zhao et al. 2023), *plumbago auriculata* NC041245 (Yao et al. 2019), *plumbago zeylanica* OR712438 (in this study), *limonium bicolor* NC059915 (Darshetkar et al. 2021), *limonium aureum* NC045399 (Zhang et al. 2020), *limonium sinense* MN599096 (Li et al. 2020), *limonium otolepis* NC065861, *rheum alexandrae* MZ997426 (Zhang et al. 2022b), *polygonum aviculare* OK661156 (Zhang et al. 2022a).

The short reads coverage depths of the assembled genome can be seen in Figure S1. The length of the complete chloroplast genome sequence of *P. zeylanica* was 169178 bp, with a large single copy (LSC) region of 92135 bp, a small single copy (SSC) region of 13455 bp. and two separated inverted repeated (IR) regions of 31749 bp (Figure 2). A total of 124 genes were identified in the chloroplast genome of *P. zeylanica*, including 79 protein-coding genes, 37 tRNA genes and eight rRNA genes. Thirteen genes are cis-splicing genes which contain introns (Figure S2), *rps*12 is a trans-splicing gene (Figure S3). In IR regions, 18 genes were detected, including *ycf*1, *trn*N-GUU, *trn*R-ACG, *rrn*5, *rrn*4.5, *rrn*23, *trn*A-UGC, *trn*I-GAU, *rrn*16, *trn*V-GAC, *rps*7, *ndh*B, *trn*L-CAA, *ycf*2, *trn*I-CAU, *rpl*32, *rpl*2, *rps*19. The overall GC content was 37.2%, and the corresponding contents for LSC, SSC and IR regions were 35.1%, 31.7% and 41.3%, respectively. We detected 84 SSR markers ranging from mononucleotide to trinucleotide repeat motif. The Phylogenetic results demonstrated a close relationship between *P. zeylanica* and *P. auriculata* in the *Plumbago* clade (Figure 3). The complete plastome sequence of *P. zeylanica* will not only provide effective use of this species, but also for the phylogenetic studies of Plumbaginaceae.

## 4. Discussion and conclusion

In this study, we sequenced and assembled the complete chloroplast sequence of *P. zeylanica.* We also elucidated all the genes in the complete chloroplast genome of species. *Plumbago* comprise medicinally important species around the world, the first complete chloroplast genome report of *P. zeylanica* providing an opportunity for further research on the species and *Plumbago*. This powerfully molecular genetic markers can be used to explore the genetic diversity, and would be also helpful in the species identification and conservation in genetic level. Phylogenetic analysis revealed a sister relationship between *P. zeylanica* and *P. auriculata*. The chloroplast genomes of the two species displayed similar gene content and gene order. *Plumbago* species are mainly distributed in warm tropical regions with approximately 20 species (Sandeep et al. 2011; Koutroumpa et al. 2018). In this genus, only one species’ chloroplast genome has been reported in previous research (Yao et al. 2019). To have a better understanding the relationships between species and detect the chloroplast genome structures features in *Plumbago*, we should conduct more extensive sampling. More species’ chloroplast genomes need to be sequenced and assembled in the future research.

## Supplementary Material

Supplemental Material

Supplemental Material

Supplemental Material

Supplemental Material

## Data Availability

The genome sequence data supporting the funding are available in the GenBank of NCBI at https://www.ncbi.nlm.nih.gov/ under accession number OR712438. The associated BioProject, SRA, and Bio-Sample numbers are PRJNA1031776, SRR26560008 and SAMN37959988, respectively.
