# Supplementary figures and images for: The complete chloroplast genome of the medicinally important plant Plumbago zeylanica L. (plumbaginaceae) and phylogenetic analysis
Source: Mitochondrial DNA B Resour. 2024 Apr 3;9(4):428–31. doi: 10.1080/23802359.2024.2333574 (PMC10993748; doi:10.1080/23802359.2024.2333574)

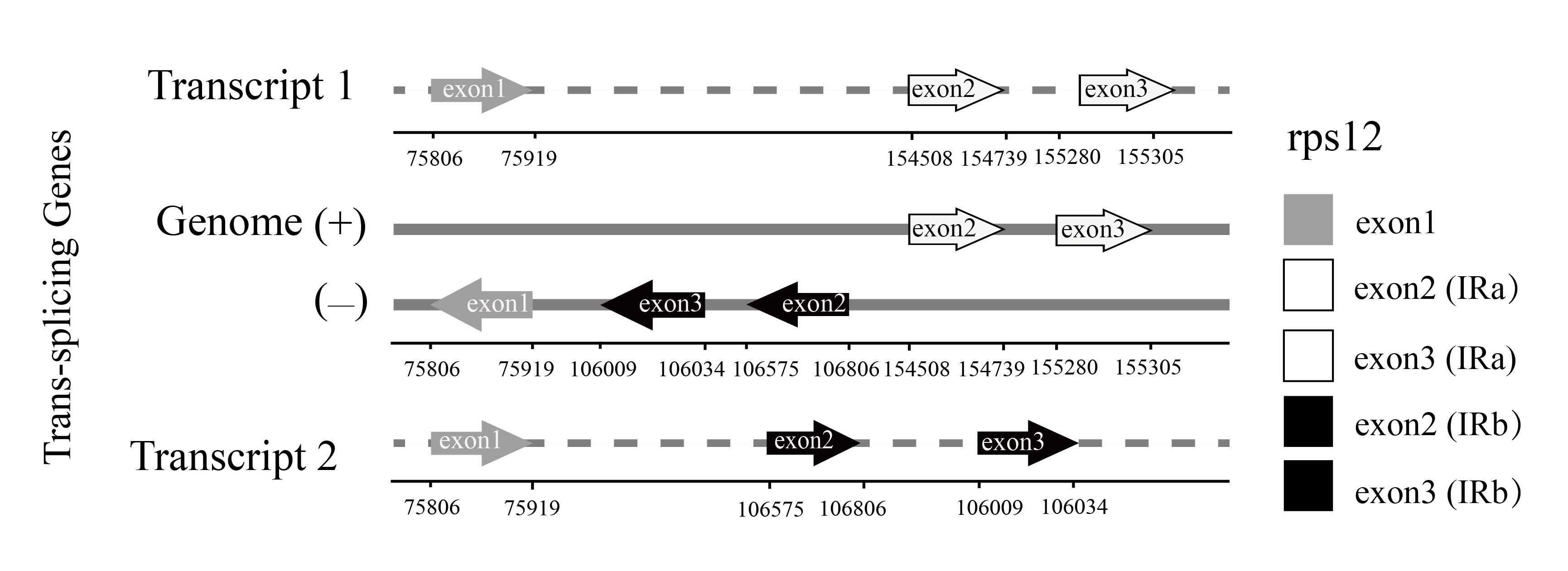

Supplement: Supplemental Material [file TMDN_A_2333574_SM4595.jpg]

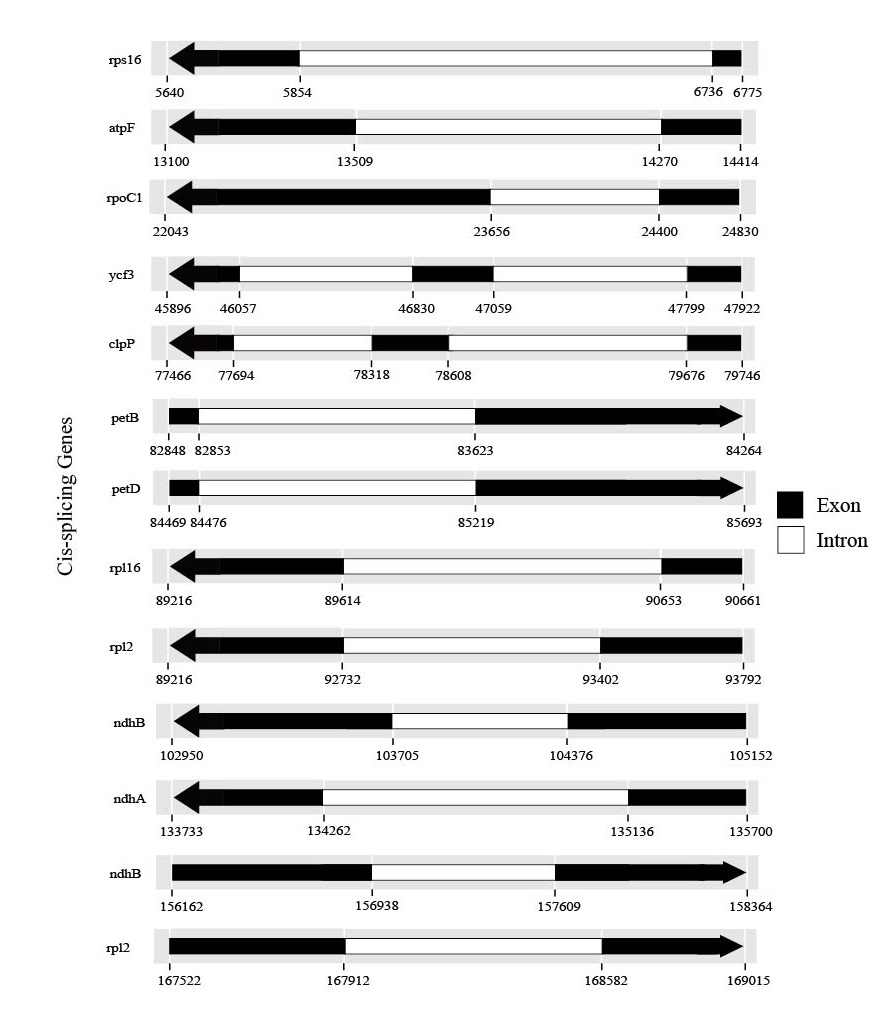

Supplement: Supplemental Material [file TMDN_A_2333574_SM4594.jpg]

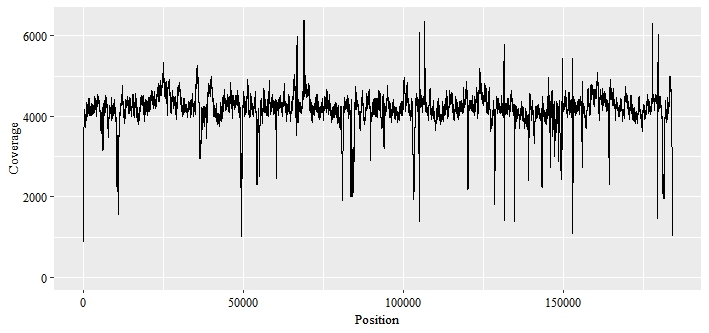

Supplement: Supplemental Material [file TMDN_A_2333574_SM4593.jpeg]
