# Supplementary material for: The complete chloroplast genome of the medicinally important plant Plumbago zeylanica L. (plumbaginaceae) and phylogenetic analysis
Source: Mitochondrial DNA B Resour. 2024 Apr 3;9(4):428–31. doi: 10.1080/23802359.2024.2333574 (PMC10993748; doi:10.1080/23802359.2024.2333574)
Supplement: Supplemental Material [file TMDN_A_2333574_SM4588.docx]

**Supplemental material**

**Figure S1**. The sequencing depth of coverage plots of *Plumbago zeylanica*. The Illumina sequences were mapped to the chloroplast genome of *P. zeylanica* in Geneious software. We exported the coverage information and draw the line chart. The horizontal axis represents the length positon of genome, while the vertical axis represents the coverage depth.

**Figure S2** Schematic map of cis-splicing genes of *P. zeylanica*. Genes were arranged based on the order in the chloroplast genome. Gene names were presented on the left, the structures were presented on the right. Exons were shown in black, intros were shown in white. The arrows represent the directions of genes. The length of exons and introns were not drawn to scale.

**Figure S3** Schematic map of trans-splicing gene *rps12* of *P. zeylanica*. The gene comprises three unique exons, two of them in the IR regions are duplicated.
